# Enhancing Environmental and Human Health Management Through the Integration of Advanced Revitalization Technologies Utilizing Artificial Intelligence

**DOI:** 10.3390/toxics12120847

**Published:** 2024-11-25

**Authors:** Mirela Volf, Ante Vučemilović, Željko Dobrović

**Affiliations:** 1 The Department of Branch Tactics, Croatian Military Academy “Dr. Franjo Tuđman”, 10000 Zagreb, Croatia; volf.mirela@gmail.com; 2 The Dean’s Office, Defense and Security University “Dr. Franjo Tuđman”, 10000 Zagreb, Croatia

**Keywords:** contaminants, ecosystem equilibrium, compromised physiological condition, artificial intelligence (AI)

## Abstract

Pollution can be broadly defined as the presence of contaminants or energy sources detrimental to ecosystems and human health. The human organism serves as a valuable indicator of ecosystem contamination. However, understanding physiological disorders and correlating specific contaminants with disease development is a complex and arduous task, necessitating extensive scientific research spanning years or even decades. To facilitate a more rapid and precise understanding of the physiological impairments induced by various contaminants, a comprehensive approach is indispensable. This review proposes a model for such an approach, which involves the systematic collection and analysis of data from ecosystem contamination monitoring, integrated with biomedical data on compromised physiological conditions in humans across different temporal and spatial scales. Given the complexity and sheer volume of data, alongside the imperative for strategic decision-making, this model leverages the capabilities of artificial intelligence (AI) tools. Although this paper exemplifies the model by investigating the effects of contaminants on the human organism, the model is adaptable to all ecosystem components, thereby supporting the conservation of plant and animal species.

## 1. Introduction

Pollution is generally defined as the introduction of harmful substances or energy into ecosystems and the human body. The causes of pollution can be anthropogenic or attributable to natural forces and disasters. Given the advanced state of technological development and the scale of the global population, current levels of global pollution are regarded as the most intense in recorded history [1]. Various spectra of pollutants present in the air, water and soil exert negative effects on human health, plant, and animal ecosystems. These substances can induce respiratory illnesses such as asthma and bronchitis due to particulate matter pollution (PM2.5 and PM10), as well as infectious diseases like dysentery or cholera. Water pollution can lead to neurodegenerative diseases, while the accumulation of toxins, pesticides, and heavy metals in the food chain via soil contamination diminishes food security [2,3]. Environmental and human health management, regarded as distinct domains in scientific and societal contexts, are confronted with the emergence of new pollutants and consequent novel diseases. Traditional approaches often prove inadequate in addressing complex challenges such as the persistent contamination of extensive areas, the degradation of entire ecosystems, and public health risks linked to environmental factors. One of the most effective strategies to advance environmental management involves the application of contemporary environmental revitalization technologies utilizing AI. Modern AI technologies facilitate the analysis of extensive datasets, recognition of historical pollution trends, and decision-making through predictive modeling for remediation efforts. This approach significantly enhances resource management efficiency and mitigates adverse environmental impacts [4]. The integration of artificial intelligence and advanced environmental revitalization methodologies, including optimized natural bioremediation processes, presents a promising framework for developing future pollution mitigation strategies, restoring ecosystems and safeguarding the health of humans, flora, and fauna [5,6]. Reliable AI models are already being applied in environmental science to address specific challenges, such as data collection and analysis from diverse sources, and the development of models for predicting climate change and natural disasters [7]. Conversely, AI tools are being extensively applied in the public health and biomedical sectors. Health index data are aggregated from sources such as the Web of Science and various Google surveys conducted by numerous administrative entities [6]. Thus, AI has become an integral tool in modern clinical decision-making processes and the implementation of personalized therapeutic interventions [6,8,9]. Research in practice has shown that challenges in personalized treatment approaches for oncology patients are better addressed using AI machine learning algorithms than with traditional methods for prognosis and therapy prediction [10]. AI tools also enable disease spread prediction and faster response for preventive and prophylactic measures, as demonstrated in research using the HealthMap system during the coronavirus pandemic [11]. The integration of AI into various social, technical, and natural sciences has demonstrated clear positive outcomes. This shift has streamlined human activity by reducing redundant and unnecessary data in business processes, allowing for more rapid and precise achievement of targeted results through advanced technologies. A key advantage is the speed at which large volumes of data can be processed. Although there is a prevalent fear within the population regarding potential control by AI systems, its targeted and regulated use has undeniably enhanced quality of life. However, obstacles to the application of AI technologies in biomonitoring and biomedicine are primarily linked to time constraints and capacity limitations for sorting and analyzing extensive datasets, as well as a shortage of skilled personnel equipped to manage complex processes and data [8,9,12]. Achieving sustainable ecosystem development and enhancing human health requires a synergistic, integrated and comprehensive approach. Accordingly, this review article aims to present a holistic model for advancing environmental and human health management through the integration of modern revitalization technologies and AI tools.

## 2. Effects of Pollution on the Environment and Human Health

### 2.1. Modern Perspectives on Environmental Pollution

Environmental pollution is recognized as the most pressing challenge in modern society. It involves the introduction of harmful substances into the natural environment, exerting adverse effects on the quality of human life and the biodiversity of ecosystems [13]. Through technological advances and comprehensive anthropogenic activities, various environmental components are subjected to temporary or permanent pollution. Moreover, population growth and urbanization processes promote the intensive exploitation of natural resources [14]. In middle- and high-income countries, legal regulations constrain the discharge of pollutants into the environment without prior treatment. Conversely, in low-income countries, economic constraints, poverty, and inadequate implementation of environmental protection laws often lead to non-compliance. Consequently, the concept of transboundary movement of pollutants emerges as a global issue. Through various environmental pathways, particularly air and surface or groundwater routes, pollution originating in one country migrates and contributes to environmental challenges in other nations [15,16]. The destabilization of ecosystems due to anthropogenic activities can result in the emergence of diseases and elevated mortality rates among plant and animal species [17]. These significant impacts of pollution are influenced by social determinants such as educational attainment and income levels. In low-income countries, population priorities typically focus on securing access to food and shelter, often relegating concerns about escalating environmental problems to a secondary position [18]. The pathways for the dissemination of pollutants from waste into the environment encompass air, water, soil, and vegetation. Pollution effects are evident through physiological and behavioral alterations in organisms. Birds and higher marine organisms are notably at risk, exhibiting heightened mortality rates from entanglement and the ingestion of fibrous materials. Moreover, pollutants undergo fragmentation or degradation over time, accumulating through the process of biomagnification in the food chain. This process entails genotoxic and mutagenic consequences, particularly impacting the progeny of specific species [19,20]. The synergistic effects of various environmental stressors, including pollutants, pathogens, and other factors, are well documented as multifactorial biotic and abiotic stressors on organisms. These stressors, often associated with global warming and climate change, encompass drought periods, fluctuations in soil carbon and nitrogen levels, and the presence of microplastics, pharmaceuticals, heavy metals, high salinity, and agricultural pesticide applications. These findings highlight their detrimental impact on sustainable environmental management practices and the biodiversity of microbial communities [18,21]. Modern science and society are advancing and recognizing the significance of integrating ecological principles into supply chains. Green Supply Chain Management (GSCM) entails the strategic selection of raw materials, product design, and manufacturing processes to incorporate environmental considerations. It also involves implementing reverse logistics to manage the product life cycle effectively. This concept is closely intertwined with Life Cycle Assessment (LCA) and the overarching principles of the Circular Economy [22,23,24].

### 2.2. Air, Water, and Soil Pollution

Air pollution is defined by the presence of pollutants in the atmospheric air that adversely affect the ecosystem and diminish its quality due to their concentration. This phenomenon has direct and indirect impacts, including contributions to global warming and ozone layer depletion. The characteristics, distribution, and effects of air pollutants are heavily dependent on their type, concentration, and atmospheric conditions. The chemical properties of these pollutants, such as oxidation/reduction potentials, solubility, and photocatalytic activity, play a crucial role in determining the extent of ecological damage [13,25]. Primary categorizations of air pollutants include carbon monoxide and dioxide, nitrogen and sulfur oxides, ground-level ozone, and fine particulate matter of 2.5 and 10 μm in size [26]. Higher concentrations of these pollutants are prevalent in urban and industrial areas of developing countries. Uncontrolled urbanization, coupled with industrialization and insufficient information on sustainable environmental management, results in significantly low air quality in these regions. Air pollution spreads to other environmental compartments such as water and soil through processes like deposition and precipitation, amplifying environmental contamination [26,27].

Water, the most precious natural resource, is profoundly affected by the ecological problem of pollution. The primary causes contributing to its deterioration include intensified industrialization, urbanization, nutrient enrichment, inadequate waste disposal practices, oil spills, and acid rain, among others. According to current research, 75–80% of water pollution stems from the discharge of municipal and sewage wastewater into the natural environment [28]. Different pollutants found in wastewater, including polycyclic aromatic hydrocarbons, pesticides, and heavy metals, represent health risks to human health. These substances have been linked to conditions such as cancer, hormonal imbalance, reproductive dysfunction, and permanent damage to internal organs [13]. Various pathogenic microorganisms present in such compromised systems lead to diseases such as cholera, typhoid fever, infectious hepatitis, dysentery, and giardiasis [29]. Water pollution can be classified into several categories based on the type of contaminants present. Physically contaminated water is characterized by changes in color, taste, and odor due to dissolved organic and inorganic substances. Chemically contaminated water results from the presence of dissolved salts, dyes, metals, pharmaceuticals, pesticides, or fertilizers. Additionally, water can become contaminated by biopollutants, which include pathogenic microorganisms from human and animal excreta, as well as radionuclides originating from nuclear facilities [29,30].

Soil pollution involves the introduction of contaminants that adversely affect soil fertility and structure, posing significant risks to both ecological systems and human health. Moreover, pollutants can migrate from soil into surface and groundwater, air, and agricultural crops, resulting in extensive and detrimental effects. This process contributes to biodiversity loss, threatening natural habitats and the survival of plant and animal species [31]. The discharge of pollutants through direct or indirect pathways originates from various waste materials from industrial facilities, hospitals, mining and processing industries, waste disposal sites, or urban environments [32]. Soil contamination encompasses a range of chemical pollutants, including pesticides and herbicides used in agriculture, synthetic fertilizers, heavy metals, and organic compounds released from industrial activities. Additionally, contamination can arise from fuels and oils associated with oil wells and point sources, as well as the presence of micro- and nanoplastics [33,34]. Table 1 presents pollutants found in environmental components along with their sources and concentrations.

### 2.3. The Impacts of Pollution on Human Health

**Figure 1:**
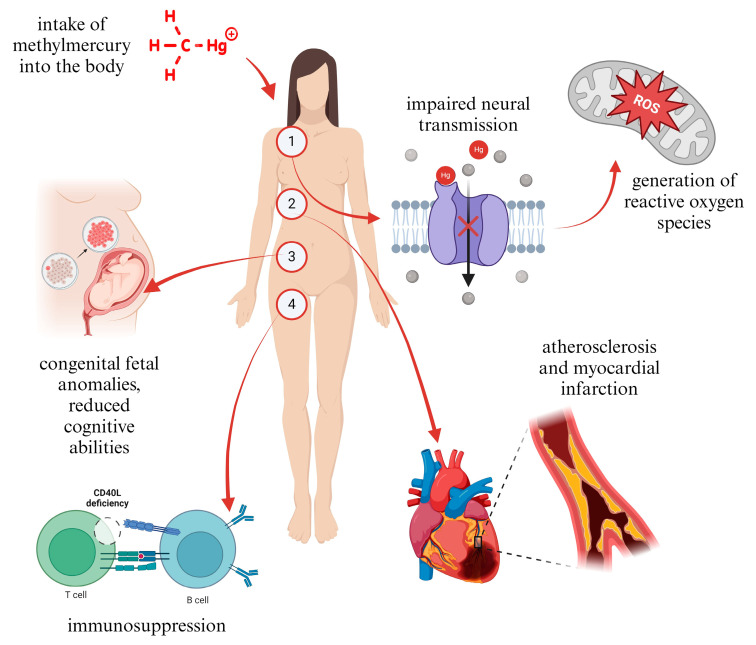
Four potential pathways of methylmercury’s effects on the human body.

Environmentally harmful technologies, particularly those failing to meet minimum ecological standards, represent a primary source of highly toxic pollutants. The quantity and diversity of these substances continue to increase spatially and temporally. This scenario poses significant challenges to biomedical sciences in understanding the biochemical mechanisms of action of such substances within the human body. The critical issue lies in detection and diagnosis, as individuals are exposed to multiple pollutants, resulting in synergistic toxic effects. Accurate diagnostics are essential for implementing targeted and personalized therapies effectively. Therefore, data collected through monitoring efforts are invaluable for both scientific research and public health initiatives [8,9]. In order to demonstrate the functionality of this model aimed at improving environmental management and human health, we will examine the example of environmental contamination by methylmercury. Mercury occurs naturally in trace amounts in the environment. However, anthropogenic activities release significant quantities of mercury into the environment, resulting in widespread pollution. Mercury is an exceptionally toxic metal with well-documented adverse health effects. It is recognized as a primary environmental pollutant, extensively utilized by humans for centuries across several sectors including agriculture, industry and medicine [46,47]. The aforementioned activities generate and release significant quantities of organic mercury. Organic mercury compounds are formed through the combination with carbon, resulting in methylmercury and ethylmercury [48,49]. The high toxicity of methylmercury presents a significant environmental concern. In aquatic ecosystems, methylmercury bioaccumulates throughout the food chain, ultimately reaching aquatic organisms consumed by humans. Severe cases of methylmercury poisoning are well documented and characterized, notably associated with the initial outbreak known as Minamata disease. Sublethal poisoning at lower levels presents a diagnostic challenge due to the gradual accumulation of mercury over prolonged periods, leading to cumulative effects [50]. At a systemic level within the body, mercury is recognized as neurotoxic, primarily impacting the nervous system. The central nervous system exhibits heightened susceptibility to the detrimental effects of methylmercury and ethylmercury, evident through clinical symptoms and histopathological changes observed in poisoned individuals. These compounds induce lesions in both the central and peripheral sensory nervous systems. Exposure to methylmercury manifests in dysfunction across numerous neurodevelopmental and neurobehavioral processes [51]. At the molecular and cellular levels, investigations conducted in both in vitro and in vivo settings have been pivotal in elucidating the molecular mechanisms underlying neurotoxicity caused by methylmercury. The primarily molecular targets of mercury compounds include thiols and selenols, especially selenoenzymes. Interactions of mercury compounds with these biomolecules result in heightened oxidative stress and the production of reactive oxygen species (ROS), which serve as triggers for apoptosis. Sublethal doses of methylmercury also have the potential to induce apoptotic cell death in cerebellar neurons [47,50]. Cellular and molecular alterations resulting from mercury exposure manifest in cytokine release, mitochondrial dysfunction, calcium imbalance, and interference with neurotransmitters such as glutamate, leading to glutamate dyshomeostasis. Upon crossing the blood-brain barrier, methylmercury induces inflammatory responses in the brain, characterized by a heightened release of prionic and inflammatory mediators from glial cells [47,51]. The specific regions of the central nervous system most affected by the direct toxicity of methylmercury are illustrated in Figure 1. Pollutants generally induce changes in the immune response of organisms, leading to immunotoxicity or immunodeficiency mediated by reactive oxygen species (ROS). In the protective mechanism, the Keap-1 sensor protein is pivotal in detecting xenobiotics within the organism, which triggers the activation of the transcription factor NRF2. NRF2 then induces the expression of detoxification genes with antioxidant and anti-inflammatory properties. This sensor response mechanism effectively protects the organism from immunotoxic effects [17,52].

## 3. Sustainable Development and Health Improvement

### 3.1. Environmentally Sustainable Technologies for Revitalization

Contemporary remediation technologies encompass methods that efficiently eliminate a wide range of pollutants from environmental matrices, generating minimal secondary waste that could negatively impact the environment. These technologies employ a synergistic combination of physicochemical and biological methods to facilitate the biotransformation and biodegradation of xenobiotics into non-toxic products and byproducts. This approach is designed to be economical, sustainable, and environmentally friendly. In the long term, it minimizes the production of harmful greenhouse gases and waste streams, thereby reducing the costs associated with additional remediation efforts [53,54]. The integration and synergy of diverse remediation techniques offer an optimal solution for achieving the desired quality and long-term benefits of environmental restoration processes. Among the naturally occurring processes, bioremediation and phytoremediation are particularly notable. These green biotechnologies are the focus of increasing scientific research and application for the oxidation, reduction, and biodegradation of various contaminants, including petroleum hydrocarbons, pesticides, pharmaceuticals, heavy metals, and other synthetic chemicals [55,56]. Phytoremediation is a biotechnology that exploits the inherent potential of plants to stabilize, absorb, or degrade various xenobiotics from contaminated soil, water, or air [57]. The biochemical mechanisms involved in this process include phytotransformation, phytostabilization, phytoextraction, and rhizofiltration [58,59]. Bioremediation has seen exponential growth over the past two decades, particularly in the areas of soil, water, and air pollution remediation. Unlike traditional physico-chemical remediation methods, bioremediation offers an alternative approach by utilizing the potential of microorganisms, including bacteria, fungi, and algae to degrade xenobiotic compounds [60]. Although bioremediation is more ecologically sustainable, certain challenges are inherent to this technology. These challenges include the duration of the biodegradation process, which is influenced by the adaptive capabilities of microorganisms and their specific growth and reproduction requirements, such as optimal temperature, pH, and nutrient availability [61]. Additionally, biodegradation can be constrained by the inhibition of enzyme activity critical for bioremediation in highly contaminated environments or the presence of specific toxic compounds that impede microbial proliferation [62]. In the context of bioremediation, enzymes synthesized by microorganisms play a pivotal role during their adaptation to pollutants. These enzymes facilitate the degradation of contaminants either intracellularly (intracellular enzymes) or extracellularly (extracellular enzymes) after being transported across the cellular membrane [63,64]. Enzymes, functioning as biocatalysts, reduce the activation energy and accelerate various biochemical reactions while remaining unchanged. To restrict enzyme mobility and ensure high catalytic efficiency, immobilization techniques are implemented. These techniques allow for the repeated use of biocatalysts, thus minimizing production or post-use filtration costs. Moreover, immobilization enhances the enzymes’ resistance to toxic environments and maintains their structural integrity and functionality during storage [65]. Enzymes that catalyze biotransformation and biodegradation processes encompass cytochrome P450, various laccases, dehalogenases, dehydrogenases, hydrolases, proteases, and lipases. Laccase enzymes are particularly notable for their capacity to reduce oxygen while oxidizing a broad spectrum of aromatic and other pollutant compounds via single-electron transfer. These laccase enzymes are produced by specific microorganisms, including *Pseudomonas putida* F6, which degrades synthetic dyes; *Streptomyces cyaneus*, which oxidizes the micropollutant bisphenol A; and *Bacillus safensis*, which decolorizes commercial dyes. The enzyme cytochrome P450 mediates electron transfer and catalysis through the oxidation/reduction of heme iron, thereby facilitating the degradation of steroids, fatty acids, and xenobiotics. This enzyme is synthesized by microorganisms such as *Rhodococcus rhodochrous*, which is involved in the degradation of hexogen, and *Bacillus megaterium*, which is responsible for the hydroxylation of polychlorinated dioxins. Dehalogenase enzymes cleave carbon-halogen bonds through three distinct mechanisms. Microorganisms producing this enzyme include *Pseudomonas* sp. TL and *Ancylobacter aquaticus*, which degrade halogenated acids; *Bacillus* sp., which degrades 2,4,6-trinitrobromophenol; and *Ochrobactrum* sp., which degrades tetrabromobisphenol A [66,67]. Examples of organisms with specific affinities for particular pollutants [68,69,70,71,72,73,74] are presented in Table 2.

Bioremediation technology can be enhanced by optimizing process parameters through biostimulation. This strategy involves stimulating the metabolic activity of microorganisms involved in the biotransformation and degradation of pollutants by adjusting factors such as oxygen concentration, essential metal availability, moisture content, salinity, and nutrient ratios (C/N/P), as well as controlling the temperature and pH of the system. Additionally, it is crucial to optimize the concentration of inhibitory and toxic substances that may adversely affect microbial biomass [75,76]. Bioaugmentation, a prominent bioremediation strategy, involves the deliberate introduction of indigenous or exogenous microbial cultures, either pure or mixed, into contaminated environments to enhance the efficiency and speed of the biodegradation processes [77]. The biological inoculum used in bioaugmentation is typically propagated in controlled bioreactors before being deployed into the polluted ecosystem. In practical applications, the introduced microbial biomass must contend with various biotic and abiotic stressors, including fluctuations in temperature, variations in humidity and pH levels, and the presence of high concentrations of toxic compounds. A critical challenge in bioaugmentation is the competitive interaction with native microbial communities already present in the contaminated site. To optimize the effectiveness of bioaugmentation, it is essential to thoroughly understand the environmental characteristics of the contaminated site and to carefully manage operational parameters within the system. This approach ensures minimal negative impacts on the vitality and biodegradation capabilities of the introduced microbial consortia [78,79]. An innovative approach in the realm of protecting and stabilizing inoculated biomass for in situ and ex situ bioremediation involves the utilization of organic, inorganic, or hybrid transport matrices. This immobilization method effectively regulates microbial activity, reduces the potential for migration from contaminated sites, and enhances the bioavailability of the pollutant substrate essential for microbial growth and proliferation [80,81]. The structural adaptability of transport matrices (such as biofilms, gels, granules, and powders) facilitates their economic feasibility and commercial application across diverse states of pollutant aggregation. This versatility allows for direct implementation in contaminated environments or bioreactors, as well as integration into biofilters for the purification of water and air. These matrices are composed of carriers including natural polymers like chitin and cellulose, synthetic materials such as silica gels, minerals with significant adsorption surfaces like natural clays or zeolites, and modern composite nanomaterials [82,83,84]. Exploring alternative strategies to enhance biodegradation or innovate new biological functionalities involves synthesizing insights from functional nanomaterials and natural systems [85]. Nanobiohybrids represent composite nanoscale materials (including metal nanoparticles, carbon nanomaterials, and quantum dots) integrated with biological entities such as microbial cells, plant cells or extracts, and enzymes. These hybrids aim to synergistically improve the degradation of pollutants within ecosystems [86,87,88]. Examples of integrated biological systems employing diverse carrier matrices are detailed in Table 3.

**Figure 2:**
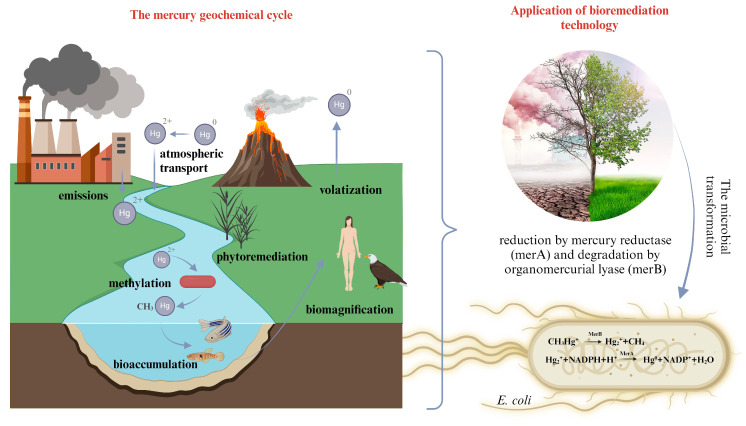
The biogeochemical cycle of mercury in nature and the application of bioremediation technology utilizing genetically modified *E. coli* in advanced microbial engineering techniques for mercury removal.

The application of hybrid technologies, such as nanobioremediation, facilitates enhanced removal of environmental pollutants by integrating nanomaterials with natural microbial degradation capabilities. Nanomaterials like carbon nanotubes (CNTs), graphene oxide, gold nanoparticles (AuNPs), silver nanoparticles (AgNPs), copper nanoparticles (CuNPs), and others, exhibit significant surface adsorption capacities, immobilizing pollutants and thereby catalyzing microbial activity [92]. Beyond microorganisms, these nanomaterials can also be synergistically combined with plant cultures to bolster plant resilience to toxic environments and expedite phytostabilization processes [93]. The aforementioned technology finds application in treating contaminated soils and wastewater, as well as in the removal of pollutants from air through integration into biofilters [94]. In addition to these methods, genetic engineering is increasingly pivotal in enhancing bioremediation effectiveness. Advances in biotechnology enable genetic modifications of microorganisms or modulation of biocatalysts to develop systems that surpass natural environmental processes in speed and efficiency [95]. Approaches such as metabolic or genetic engineering can enhance the degradation capabilities of microorganisms towards both new and persistent pollutants, improve responses to stressful conditions, or stimulate the synthesis of biologically active compounds for bioremediation applications [96]. The proposed approach has been extensively studied in the context of synthetically engineered strategies for bioremediation of Hg(II) using genetically modified *E. coli* [97]. By incorporating the mer operon (consisting of merA, merB, and transporters merT, merC, and merP), natural gene clusters found in bacteria capable of complexing and reducing mercury, *E. coli* can effectively convert toxic Hg(II) ions into less harmful elemental mercury [98]. Boyd et al. [99] examined the efficacy of genetically modified *Deinococcus geothemalis*, which, after introducing the mer operon, demonstrated a successful reduction of mercury ions in thermal environments. Figure 2 illustrates the biogeochemical cycle of mercury in nature and the application of bioremediation technology involving the mechanism of action of genetically modified *E. coli* for the reduction and attenuation of toxicity.

### 3.2. Personalized Treatment Approach and Revitalization

In biomedicine, foundational AI algorithms have been developed to support advanced technologies aimed at refining the assessment of disease risk, etiology, and outcomes. This progress has facilitated the emergence of a patient-centered approach, termed personalized medicine, which tailors treatment strategies to the unique characteristics of each individual patient [8,9,12]. The personalized treatment approach in the proposed model is grounded in a comprehensive strategy that leverages data from diagnostic methodologies and field biomonitoring. The aggregated data is systematically integrated, sorted, filtered, and benchmarked against current scientific knowledge to devise an appropriate personalized therapeutic and organism revitalization regimen. Once specific directives for personalized treatment and revitalization are established through this model, artificial intelligence algorithms can be employed to identify optimal interventions. These interventions must be holistic, encompassing both detoxification and revitalization methodologies utilizing natural and synthetic compounds. It is pertinent to highlight the instance of mercury poisoning, particularly with organic compounds like methylmercury. The natural detoxification and revitalization of the organism are most effective with enzymes such as glutathione S-transferase, which catalyzes the conjugation of glutathione with methylmercury, forming a less toxic complex that facilitates excretion [100]. Furthermore, metallothionein, a cysteine-rich protein, can bind to methylmercury, thereby mitigating its toxicity and promoting its elimination from the body [101]. The mitigation of reactive oxygen species (ROS) in cells, induced by the presence of methylmercury, is facilitated not only by antioxidant enzymes but also by non-enzymatic antioxidants such as vitamins C and E, and selenium [102]. In contrast, synthetic detoxification agents for mercury operate through chelation, forming stable complexes that are easily excreted from the body. Notable examples of these chelating agents include dimercaprol (BAL) and dimercaptosuccinic acid (DMSA). These compounds bind to methylmercury via thiol groups, enabling its mobilization from tissues and promoting its renal excretion [103]. In addition to the aforementioned compounds, N-acetylcysteine (NAC) serves as a modality for revitalization by augmenting intracellular glutathione levels, crucial for mitigating glutamate dyshomeostasis triggered by mercury toxicity. Various types of zeolites can also be utilized for their adsorption properties and buffering capacity within the organism. These natural and synthetic detoxification and revitalization agents are applicable across other heavy metal poisoning scenarios. Their optimal combinations, therapeutic dosages and treatment durations are determined through a personalized approach [104,105].

## 4. A Comprehensive Model for Enhancing Environmental and Human Health Management Through the Integration of Modern Technologies with AI

**Figure 3:**
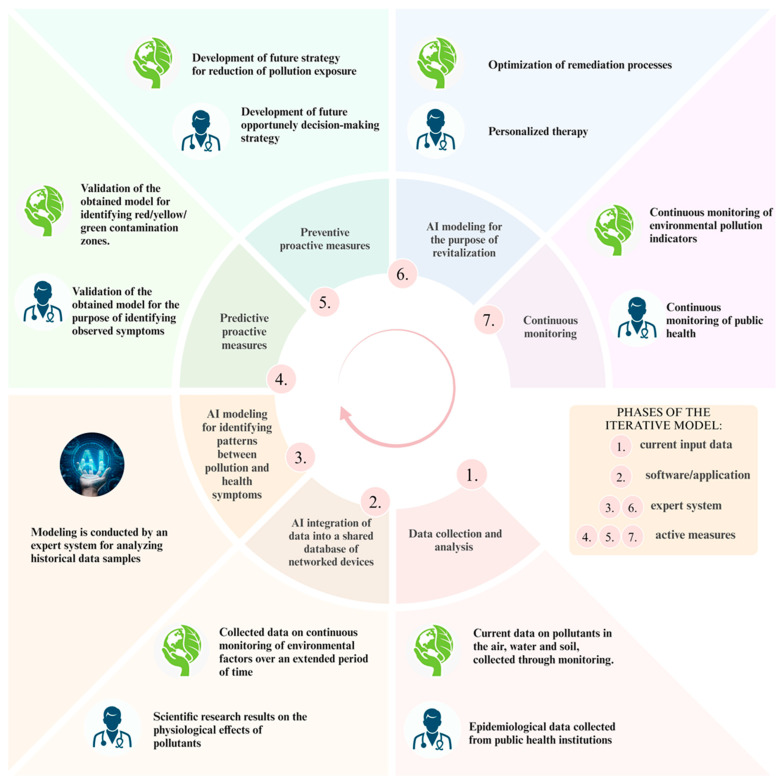
Comprehensive model of environmental and human health management: integration with AI.

This review presents a theoretical concept for a model supported by AI tools, designed to improve environmental and human health management in the event of intentional or accidental incidents. The proposed model adopts a comprehensive approach to data collection, divided into two primary categories: environmental monitoring data (concerning the presence of pollutants), and epidemiological data from public health institutions (detailing the impact of pollutants on human health). To facilitate the integration of conventional scientific methods, including sample data collection, data analysis, and medical diagnostics for clinical decision-making strategy, the AI-supported model is structured into seven distinct stages. Within these stages, except for Stage 3, two specific steps are clearly defined for both environmental and human health management (Figure 3).

### Description of the Individual Phases

In Phase 1, data on the presence of pollutants in the air, water, and soil are collected through environmental stationary monitoring stations or periodic sampling. The collected data, used for further analysis and modeling, encompass environmental factors such as concentrations of volatile organic compounds (VOCs), PM10 and PM2.5 particulates in the air, concentrations of heavy metals, pesticides, and organic compounds in soil, as well as specific biological parameters, such as the presence of coliform or pathogenic microorganisms in water. As a consequence of pollutant presence, health-related data may indicate respiratory anomalies, cardiovascular issues, toxic concentrations of heavy metals in blood and urine, food poisoning, increased rates of genotoxic mutations, dermatological inflammations, and other health conditions. These collected input data are compiled into separate databases and analyzed using comparative methods (compliance analysis) against permitted reference values for each pollutant, with deviations tracked in real-time. Simultaneously, epidemiological data are gathered for the population residing in the area under investigation. If a correlation between physiological changes in humans and environmental pollution is suspected, relevant data from environmental monitoring are isolated for further analysis.

Phase 2 introduces the first step in integrating AI tools into the proposed model. A specially designed software, managed by the AI tool, creates a unified database that connects data obtained from environmental monitoring and epidemiological samples over an extended period (from previous measurement and monitoring analyses). Using time series analysis, the AI tool identifies long-term patterns of pollution impacts on the environment and human health. This step involves detecting anomalies through deviation analysis and verifying compliance with reference values from scientific toxicological and ecotoxicological studies. The AI tool thoroughly searches large databases over an extended timeframe, thus accelerating sample analysis and minimizing human error.

Phase 3 employs an advanced AI tool, known as an expert system, to model heterogeneous real-time databases (from Phase 1) and long-term environmental and health parameter monitoring data (from Phase 2). Based on this data, the expert system quickly identifies correlations between the presence of specific pollutants in the environment and symptomatic diseases. In practice, there are already computational systems in place that assist users in solving complex tasks, recognizing patterns, and diagnosing problems, thereby supporting the decision-making process.

Phase 4 involves predictive proactive measures that scientific experts in specific fields of activity must implement. Environmental scientists validate the model’s output and identify contamination zones using Geographic Information Systems (GIS) and data analysis. Based on symptoms and the developed AI model, healthcare personnel then confirm the disease and its underlying cause.

Phase 5 represents the step of preventive proactive measures and future strategies aimed at reducing the potential harmful impact of such pollutants on the environment and human health.

Phase 6 focuses on the modeling of AI expert systems to select appropriate environmental revitalization processes and personalized therapies for affected patients. The role of AI is to model the best active measures for the remediation of polluted environments based on input data (such as the type of pollution, the components of the affected environment, and the suitable physical-chemical remediation methods or natural bioremediation processes). Additionally, using databases on the types of pollution and their physiological effects on the human body, the AI system proposes the most optimal solution for personalized patient therapy.

Phase 7 involves active measures for the continuous monitoring of pollution indicators and health data of the population through public health institutions, allowing for iterative adjustments to the model to meet future needs.

The proposed model, due to the complexity of heterogeneous databases, faces several challenges and limitations. The intricate design of expert systems in Phases 3 and 6 would incur development costs and require complex algorithms that contribute to computational power. Additionally, the input data on pollutant concentrations and their physiological effects on human health contain varying measurement units, which complicate interpretation. Before the implementation of AI expert systems for modeling, careful preparation, filtering, and preprocessing of data are essential to ensure relevance and accuracy for analysis. Despite thorough data preparation, after obtaining the model from Phases 3 and 6, it is necessary to validate its reliability through testing in real-world conditions. A common issue is the problem of non-reproducibility of the model due to inaccuracies in the programming code or small changes that can lead to errors in the final analysis. These limitations manifest in the inability to reconstruct the original models in other studies using the same input data and parameters. To further optimize the proposed model, it is essential to develop a universal approach for environmental and human health revitalization in cases of the presence of the same pollutant. Relevant input data on the presence of, for example, MeHg, modeled within AI expert systems, will enable the creation of a unified model for environmental revitalization and personalized treatment. This model must be representative and effectively present a strategic plan of active measures for environmental remediation and treatment in every future case of MeHg presence in the natural environment.

## 5. Discussion

Environmental pollution stemming from anthropogenic activities has persisted since the dawn of human civilization but intensified notably during the Industrial Revolution. This pollution is responsible for a majority of contemporary diseases as humans ingest substances that disrupt physiological balance. Despite community efforts to reduce pollution to sustainable levels through various circular and revitalization processes, a definitive solution remains elusive. Conversely, natural sciences and medicine, through their scientific advancements in toxicology, personalized treatment approaches and formulation of clinical decision-making strategies, continue to encounter substantial challenges in the prevention, diagnosis, therapy, and detoxification of the human organism. Thus, understanding the connection between environmental pollution and human health is pivotal for comprehending the impacts of pollutants on the human body. In principle, the health quality and state of the human organism serve as robust indicators of environmental pollution, yet this link encounters significant challenges due to the varied types of pollutants present over time and space, which can often exert similar physiological effects. Consequently, understanding the complex and intricate effects of pollution on human health requires extensive scientific research spanning years or even decades. While an ideal laboratory model for studying the impacts of diverse pollutants on human physiology would involve humans, ethical considerations preclude such an approach. On the other hand, animal models frequently demonstrate physiological reactions that diverge from those observed in the human body, thereby rendering them inadequate for understanding human physiology. A fundamental challenge in understanding diseases induced by environmental pollution and their effects on human health lies in the absence of systems capable of systematically gathering, analyzing, integrating, and comparing data from environmental monitoring across spatial and temporal dimensions with epidemiological profiles, disease classifications, and biomedical advancements. Due to the complexity and volume of data derived from environmental monitoring, scientific findings regarding the impacts of pollution on human physiology, and epidemiological health data, it is imperative to establish a model that integrates these elements. This integration aims to enhance environmental management and human health outcomes. The proposed iterative model outlines steps for ongoing environmental monitoring in conjunction with medical assessments essential for understanding and correlating disease causation and its effects on human physiology (Figure 3). The integration of extensive spatial and temporal environmental data with epidemiological health data requires a sophisticated model capable of managing, analyzing, and correlating these datasets effectively. Traditional analog and basic digital systems are insufficient for the complex tasks of data filtering, sorting, and comparison, essential for understanding and managing sustainable ecosystem controls and addressing diverse health challenges. Advanced computational frameworks are necessary to enable a rapid response to environmental events, facilitate comprehensive data integration, and offer diverse solutions for enhancing environmental revitalization and health preventive measures. AI has made significant advancements across a range of fields, including social sciences, technical disciplines, and natural sciences. Despite general concerns and fears regarding AI’s potential to control human activities, its targeted application has undeniably improved quality of life. The proposed model for monitoring ecosystems and human health is innovative in two key aspects: it synthesizes environmental sciences with biomedicine and integrates AI to support these processes, which would be unfeasible without such technology. To ensure the model functions effectively, it is essential to establish precise algorithms that enable AI to operate efficiently as a tool. It is crucial to emphasize that without well-defined algorithms, the model may encounter limitations, particularly in phases 3 and 6. Figure 3 delineates the phases in which artificial intelligence is engaged, emphasizing the necessity of maintaining human oversight throughout the system. Although this model is illustrated within the context of pollutant impacts on human health, its comprehensive framework is broadly applicable to all components of ecosystems and food chains. The proposed model could also be applicable for analyzing the effects of environmental pollution on animal health. In this case, the input data would include environmental monitoring data and biomonitoring samples from animals. The dataset used in this model could encompass the monitoring of histological changes in animals, increased concentrations of organic compounds in adipose tissue, genotoxicity indicators, reduced enzymatic activity, oxidative stress markers, and similar parameters. By putting this model into operational function, it could also be useful for predictive purposes. Specifically, all the data available to the AI can be utilized for predicting events and ecological risks associated with various chemicals.

## 6. Conclusions

The contemporary concept of environmental pollution involves the anthropogenic introduction of harmful pollutants into environmental components such as air, water, and soil. Due to advanced technological development, the level of pollution today is higher than ever in human history. Consequently, synergistic effects of various pollutants on the human body emerge, complicating accurate diagnosis and treatment. Modern environmental revitalization technologies, such as bioremediation, biostimulation, bioaugmentation, the use of nanobiohybrids, and nanomaterials, combined with personalized, holistic approaches to human health treatment, represent significant potential for integration with AI tools. The proposed comprehensive model includes a strategy for improved management of the environment and human health when integrated using AI tools. This model encompasses the collection, filtering, processing, comparative analysis, and modeling of data sets from environmental monitoring and health data regarding the effects of pollution on human health. This approach aims to improve control over the sustainable development of ecosystems and provide a more thorough understanding, diagnosis, and treatment of diseases caused by pollutants.

## Figures and Tables

**Table 1:** Pollutants in different environmental matrices, their sources of origin, and the measured concentrations.

A Component of the Environment	Category of Polluting Substance	The Type of Polluting Substance	Source of Origin	Concentrations in the Environment	Reference
Air	carbon compounds	CO	industrial processes, combustion of fossil fuels, agricultural activities, oil refineries	>6 ppm	[35]
	nitrogen compounds	NO_x_	industrial processes, combustion of fossil fuels, agricultural activities	0.1 ppm	[36]
	oxygen compounds	ground ozone (O_3_)	photochemical reactions between precursors (SO_x_ or NO_x_) and volatile organic compounds	79 ppb	[37]
	tiny micron particles	PM_2.5_	combustion of fossil fuels, combustion of biomass, industrial processes, dust particles	34.7 μg/m^3^	[38]
	tiny micron particles	PM_10_	combustion of fossil fuels, combustion of biomass, industrial processes, household emissions, construction activities	45.90–77.23 μg/m^3^	[39]
Water	plasticizer for polymers and resins	diethyl phthalate	chemical industry, building materials, households	2000 ng/L	[40]
	fixative and perfume for soaps	benzophenone	chemical industry, municipal wastewater	400–16,000 ng/L	[40]
	herbicide	atrazine	leachate from agricultural activities	19–388 ng/L	[41]
	antibiotic	erythromycin	municipal wastewater, leachate from waste disposal sites	53–1060 ng/L	[41]
	phytoestrogen	beta-sitosterol	cosmetic, food and pharmaceutical industry	24,000 ng/L	[41]
Soil	heavy metals	lead	chemical industry, mining industry, waste disposal site	19.2 mg/kg	[42]
	organochlorine compounds	PCB (polychlorinated biphenyl)	industrial processes, e-waste, construction materials, landfills	166.15 pg/g	[43]
	oil derivative	diesel	accidents from oil pipelines or processing plants	20 g/kg	[44]
	radionuclide	Ra	medical waste, radioactive waste from nuclear plants	89,000 ± 9000 Bq/kg	[45]

**Table 2:** Classification of specific microorganisms involved in the degradation of particular pollutants.

Organism	Genus/Species	Affinity for Pollutants in the Environment
Bacteria	*Arthrobacter* sp.	nitrophenol
	*Exiguobacterium aurantiacum*	phenols, heterocyclic compounds, PAH
	*Ralstonia eutropha*	dichlorophenoxyacetic acid
	*Pseudomonas aeruginosa*	atrazine, phenol, Cd, Pb, Cu, Ni, Ra, Zn, crude oil, aromatic hydrocarbons
	*Pseudomonas putida*	monocyclic aromatic hydrocarbons (benzene, xylene, toluene), diesel, petrol
	*Escherichia coli*	Zn, V, Cr
	*Micrococcus* sp.	Th, U, some hydrocarbons
	*Bacillus* sp.	endosulfan
Algae	*Spirulina* sp.	Pb, Cd, Ni, pesticides, phenol, textile dyes
	*Chlorella vulgaris*	Cd, Th, Zn, Pb, Ni, Cs, tetracycline, levofloxacin
	*Monoraphidium braunii*	bisphenol A
	*Chlorococcum humicola*	Fe
	*Chlorella pyrenoidosa*	Cd, Pb, Hg, PCB, phenol, triclosan
Fungi	*Penicillium* sp.	benzo(a)pyrene, aliphatic hydrocarbons
	*Phanerochaete chrysosporium*	phenanthrene, anthracene, pyrene, fluoranthene, 2,4-dichlorophenol, DDT, ibuprofen
	*Lentinus* sp.	phenanthrene, pyrene
	*Aspergillus niger*	petroleum derivatives, diesel, Pb, Cr, Cd, Cu, dichlorfenac
	*Saccharomyces cerevisiae*	heavy metals; Ni, Hg, Pb
	*Trametes versicolor*	ibuprofen, salicylic acid, erythromycin, estriol
	*Ganoderma lucidum*	pyrene
Plants	*Phragmites australis* (*common reed*)	adsorption of organic pollutants, denitrification, phytostabilization of heavy metals (Al, Mn, Pb, Ni, Cr, Hg)
	*Pteris vittata* (fern)	phytoremediation of As (V), rhizofiltration of As (III)
	*Cannabis sativa* (indian hemp)	hyperaccumulation of organic contaminants (PAH, benzo(a) pyrene, naphthalene, chrysene) and heavy metals (Se, Co, Pb, Cu)
	*Eichhornia crassipes* (water hyacinth)	methyl blue and orange, removal of Pb, Cu, Zn, Hg, Cd, Cr and nutrients

**Table 3:** Examples of integrated biological systems utilizing composite transport carriers for the biodegradation of targeted pollutants.

Transport Carrier Type	Operation Principle of the Transport Carrier	Immobilized Biological System	Reference
synthetic polymer matrix (poly-vinyl alcohol, PVA) coating with a shell (hydrophobic poly (p-xylylene), PPX	biomimetic extracellular films (“living composites”) for nitrite bioremediation	*Nitrobacter winogradskyi*	[89]
plant fiber sponge derived from *Luffa cylindrica* or *Luffa aegyptiaca*	the polymer sponge matrix prevents the spread of biomass into the environment; design is used for the biodegradation of aromatic hydrocarbons in in situ conditions	*Bacillus cereus*	[84]
PVA, poly-vinyl alcohol/ SA, sodium alginate/bentonite bio-composite matrix	immobilization of a mixed bacterial strain in the pores of the matrix for biodegradation of Total Petroleum Hydrocarbon (TPH); large adsorption surface and stabilizing effect on microorganisms	biosurfactant-producing bacteria *Pseudomonas aeruginosa*, *Bacillus subtilis*, *Ralstonia pickettii*	[90]
carbon nanotubes (CNTs)	biodegradation of ciprofloxacin; combined effect of sorption and biological removal in anaerobic conditions using CNT and *L. portucalensis*	aerobic bacteria *Labrys portucalensis*	[91]

## Data Availability

The data presented in this study are available on request from the corresponding author.
